# Probing *Clostridium difficile* Infection in Complex Human Gut Cellular Models

**DOI:** 10.3389/fmicb.2019.00879

**Published:** 2019-04-30

**Authors:** Blessing O. Anonye, Jack Hassall, Jamie Patient, Usanee Detamornrat, Afnan M. Aladdad, Stephanie Schüller, Felicity R. A. J. Rose, Meera Unnikrishnan

**Affiliations:** ^1^Microbiology and Infection Unit, Division of Biomedical Sciences, Warwick Medical School, University of Warwick, Coventry, United Kingdom; ^2^Warwick Integrative Synthetic Biology Centre, School of Life Sciences, University of Warwick, Coventry, United Kingdom; ^3^Division of Regenerative Medicine and Cellular Therapies, School of Pharmacy, Centre for Biomolecular Sciences, University of Nottingham, Nottingham, United Kingdom; ^4^Norwich Medical School, Faculty of Medicine and Health Sciences, University of East Anglia, Norwich, United Kingdom; ^5^Gut Health and Food Safety Programme, Quadram Institute Bioscience, Norwich, United Kingdom

**Keywords:** *Clostridium difficile*, 3D gut epithelium, gut infection model, colonization, *C. difficile*–commensal interactions, vertical diffusion chamber

## Abstract

Interactions of anaerobic gut bacteria, such as *Clostridium difficile*, with the intestinal mucosa have been poorly studied due to challenges in culturing anaerobes with the oxygen-requiring gut epithelium. Although gut colonization by *C. difficile* is a key determinant of disease outcome, precise mechanisms of mucosal attachment and spread remain unclear. Here, using human gut epithelial monolayers co-cultured within dual environment chambers, we demonstrate that *C. difficile* adhesion to gut epithelial cells is accompanied by a gradual increase in bacterial numbers. Prolonged infection causes redistribution of actin and loss of epithelial integrity, accompanied by production of *C. difficile* spores, toxins, and bacterial filaments. This system was used to examine *C. difficile* interactions with the commensal *Bacteroides dorei*, and interestingly, *C. difficile* growth is significantly reduced in the presence of *B. dorei*. Subsequently, we have developed novel models containing a myofibroblast layer, in addition to the epithelium, grown on polycarbonate or three-dimensional (3D) electrospun scaffolds. In these more complex models, *C. difficile* adheres more efficiently to epithelial cells, as compared to the single epithelial monolayers, leading to a quicker destruction of the epithelium. Our study describes new controlled environment human gut models that enable host–anaerobe and pathogen–commensal interaction studies *in vitro*.

## Introduction

*Clostridium difficile*, an anaerobic spore-forming bacterium, is the main cause of infectious diarrhea in healthcare settings. A major risk factor for *C. difficile* infection (CDI) is antibiotic use, which results in the disruption of the intestinal microbiota, allowing *C. difficile* to colonize and proliferate (Adamu and Lawley, 2013; Fuentes et al., 2014). CDI is one of the most common healthcare-associated infections in the United States, with an estimated 453,000 cases of CDI and 29,000 deaths reported in 2011, and with the highest mortality rates among the elderly (Lessa et al., 2015). Although the number of cases in the United Kingdom is declining (13,000 cases in 2016–2017; Public Health England, 2017), an increasing incidence of CDI has been reported across Europe, Canada, and Australia (Gravel et al., 2009; Davies et al., 2014; Collins et al., 2017).

*Clostridium difficile* infection pathogenesis is complex and mediated by a number of bacterial virulence factors. *C. difficile* produces two main toxins, toxin A and toxin B (TcdA and TcdB), and some strains produce a binary toxin (CDT), all of which contribute to bacterial pathogenicity (Kuehne et al., 2014; Carter et al., 2015). Toxins A and B are known to disrupt the intestinal epithelial barrier function through inactivation of the Rho GTPases, which results in reorganization of the actin cytoskeleton and induction of the MAPK pathways, leading to a cytokine response (Bobo et al., 2013; Chen et al., 2015). The key role for these toxins during infection has been demonstrated in animal models using toxin mutants, which are unable to cause disease and death (Lyras et al., 2009; Kuehne et al., 2010, 2014; Carter et al., 2015). While toxins are major virulence factors, studies have highlighted the importance of several *C. difficile* surface proteins such as surface layer proteins (Calabi et al., 2002; Merrigan et al., 2013), adhesins (Hennequin et al., 2001; Barketi-Klai et al., 2011; Kovacs-Simon et al., 2014), cell wall proteins (Waligora et al., 2001), pili, and flagella in CDI (Tasteyre et al., 2001; Batah et al., 2017; McKee et al., 2018). *C. difficile* is also known to produce spores that are resistant to antibiotics and disinfectants (Lawley et al., 2010; Vohra and Poxton, 2011). *C. difficile* spores mediate disease transmission and may serve as a reservoir within the host causing recurrence of infection (Deakin et al., 2012).

Although animal models of CDI have been used to understand *C. difficile* pathogenesis and investigate the functions of several *C. difficile* factors (Chen et al., 2008; Kuehne et al., 2010; Deakin et al., 2012; Darkoh et al., 2016), it is challenging to study host–bacterial interactions occurring at the gut mucosal interface *in vivo*. *In vitro* human cell culture models enable molecular and cellular studies on both the host and the pathogen, easier testing of multiple conditions, and visualization of infection dynamics. Infection of human intestinal epithelial cell (IEC) lines has been used to study *C. difficile* pathogenesis but these models have been limited to short time periods as *C. difficile* requires an anaerobic environment for optimal growth (Cerquetti et al., 2002; Janvilisri et al., 2010; Mora-Uribe et al., 2016). A dual environment system such as a vertical diffusion chamber (VDC), which permits growth of the bacteria and IECs in appropriate gaseous environments, was used previously by Schüller and Phillips (2010) to demonstrate increased adherence of enterohemorrhagic *Escherichia coli* (EHEC) to polarized IECs in microaerobic compared to aerobic conditions, accompanied by enhanced expression and translocation of EHEC type III secreted effector proteins. Similarly, an increase in *Campylobacter jejuni* invasion of IECs was observed under a microaerobic environment in the VDC (Mills et al., 2012). Recently, a VDC was employed to culture *C. difficile* with T84 cells, and an anaerobic environment was shown to enhance *C. difficile-*induced cytokine production, compared to aerobic co-culture, although this was only studied over a short time course (Jafari et al., 2016).

Models that replicate the physiology and local tissue environment found *in vivo* are ideal for investigating pathogen interactions in IECs. Three-dimensional (3D) gut models support better epithelial cell growth and differentiation through proteins such as growth factors secreted by underlying cell layers (Morris et al., 2014; Kook et al., 2017). Scaffolds generated from natural or synthetic polymers such as matrigel, collagen, and polyethylene terephthalate (PET) have been used to generate the extracellular matrix (ECM), which is a major tissue component, and supporting scaffolds can be designed to meet the cell type-specific needs (Ravi et al., 2015; Kook et al., 2017). More recently, electrospinning has been employed to fabricate fibers from polymers creating a structure similar to the natural fibrous network of the ECM (Morris et al., 2014; Kook et al., 2017). Electrospinning was used to create nanofiber and microfiber scaffolds for optimal 3D culture of airway epithelial and fibroblast cells (Morris et al., 2014). Fibroblasts play an active role in producing ECM and producing chemokines in response to bacterial infection (Smith et al., 1997).

Three-dimensional gut models have been employed to understand key bacterial and host pathways in a range of pathogens including *C. difficile* (Kasendra et al., 2014; Leslie et al., 2015), *Salmonella* (Barrila et al., 2017), *Cryptosporidium parvum* (DeCicco RePass et al., 2017), and Coxsackievirus B (Drummond et al., 2016). In human intestinal organoids infected with *C. difficile*, bacteria were reported to be viable for up to 12 h (Leslie et al., 2015), while a 3D model using Caco-2 cells grown as cysts in a matrigel monitored the adhesion and translocation of *C. difficile* for 1 h (Kasendra et al., 2014). While both studies investigated the role of *C. difficile* toxins in these models, neither was able to follow the infection for a longer period of time due to the lack of an optimal environment for bacterial growth.

The aim of this study was to develop improved gut models to study *C. difficile*–host interactions over longer timescales. Here, we have studied the infection dynamics of *C. difficile* over an extended time frame using human gut epithelium models. Using an epithelial VDC (E-VDC) model, we demonstrate an increase in the numbers of adherent *C. difficile* accompanied by production of spores, toxins, and bacterial filamentous forms, along with host chemokine production, over 48 h. We demonstrate that this system can be used to study interactions of obligate anaerobes such as the gut commensal, *Bacteroides dorei*, with IECs. Interestingly, we show that *C. difficile* replication is significantly reduced in presence of *B. dorei* on gut epithelial cells. In a complex 3D model that we developed which contains myofibroblasts on electrospun nanofiber scaffolds, we observed increased bacterial adhesion to the IECs, compared to the E-VDC model.

## Results

### Development of an Epithelial VDC Infection Model for *C. difficile*

**FIGURE 1:**
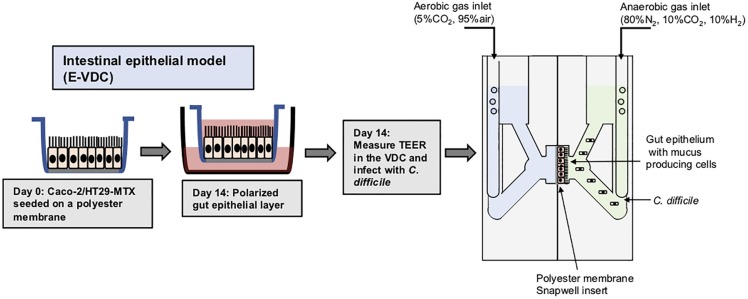
Scheme for generation of epithelial layers for use within vertical diffusion chambers. For the 2D model, intestinal epithelial cells were grown to form a polarized confluent monolayer in a Snapwell insert for 2 weeks before inserting in the VDCs. *C. difficile* was added to the apical chamber which was perfused with an anaerobic gas mix and incubated for different times while maintaining anaerobic conditions. The basolateral compartment was perfused with a mixture of 5% CO_2_ and 95% air, necessary for growth of IECs.

**FIGURE 2:**
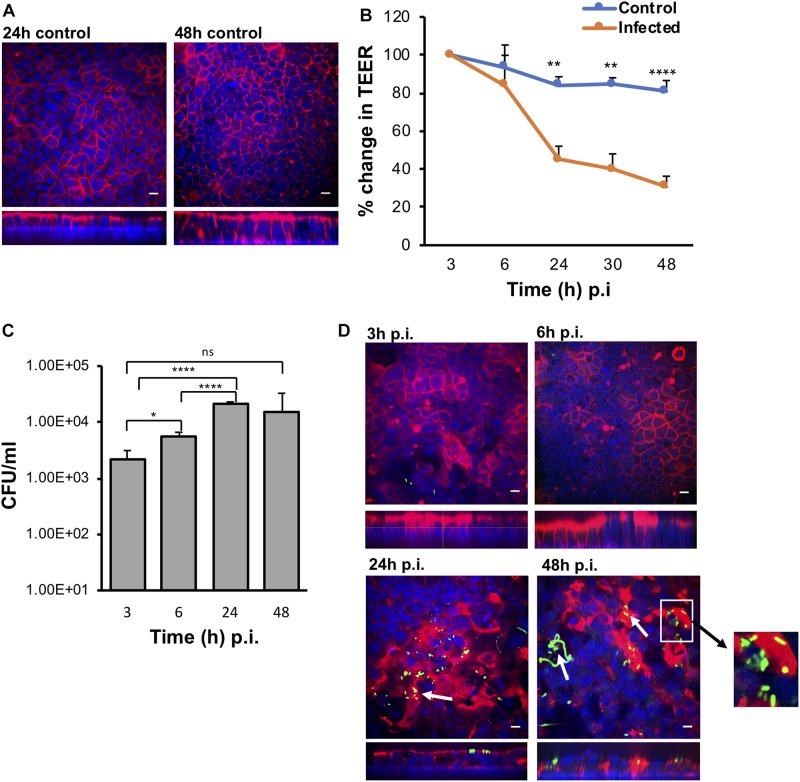
The VDC permits growth of host cells and enables *C. difficile* to adhere to gut epithelial cells. **(A)** Immunofluorescent microscopy images of uninfected controls at 24 and 48 h. Actin is stained with phalloidin (red) and cell nuclei are stained with DAPI (blue), scale bar = 10 μM. **(B)** Reduction in TEER measurements at different times after infection indicating increasing permeability compared to uninfected controls, **p < 0.01, ****p < 0.0001 as determined by one-way ANOVA. **(C)** Colony counts from infected cell lysates to determine the number of adherent or cell-associated vegetative bacteria. A significant increase in adherent *C. difficile* is observed from 3 to 24 h. Data shown are the mean of three independent experiments in triplicates and error bars indicate SD, *p < 0.05 and ****p < 0.0001 as determined by one-way ANOVA with Tukey’s test for multiple comparison. **(D)** Immunofluorescent microscopy images of *C. difficile* infected IECs at 3, 6, 24, and 48 h p.i. showing colocalization (yellow) of the bacteria stained with anti *C. difficile* antibodies (green) with actin, stained with phalloidin (red). Cell nuclei are stained with DAPI (blue). Reorganization and destruction of actin filaments is observed at 24 and 48 h p.i. Inset (white square box) shows micro-communities, and bacterial colocalization with actin at 24 and 48 h (yellow) and *C. difficile* filamentous forms observed at 48 h p.i. are indicated by white arrows, scale bar = 10 μM. Inset below shows the orthogonal *XZ* axis view of the IECs.

Polarized IECs (epithelial Caco-2 and mucus producing HT29-MTX goblet cells) were cultured on the apical side of a Snapwell insert as shown in Figure 1. Inserts with polarized cell layers were placed in the VDC and experiments were performed by perfusing a mixture of 5% CO_2_ and 95% air in the basolateral compartment for epithelial cell growth and an anaerobic gas mixture (10% CO_2_, 80% N_2_, and 10% H_2_) on the apical side for bacterial growth. After 3 h, the cell layers were washed with PBS, followed by the addition of fresh prereduced Dulbecco’s Modified Eagle Medium (DMEM) supplemented with 10% fetal bovine serum (FBS; to simulate infection conditions described below). No significant disruption to the IECs was seen after 24 or 48 h by microscopy; actin staining showed that the cytoskeleton of the control cells was intact at 24 and 48 h (Figure 2A).The barrier integrity of IECs was monitored by measuring the transepithelial electrical resistance (TEER). The TEER values demonstrated an intact epithelium in the control IECs incubated within the VDC, although a slight decline was observed over 24 h (Figure 2B). Immunofluorescent staining of the Snapwell inserts for MUC2, a major mucus protein produced by goblet cells, showed that a small amount of mucus was produced (Supplementary Figure S1) in this cell layer which contained 10% goblet cells (90% Caco-2:10% HT29-MTX).

Furthermore, to ensure that anaerobic conditions were maintained in the apical chamber, growth of *C. difficile* in the apical compartment of the VDC was compared to growth in the anaerobic cabinet for 3 and 24 h. No negative impact on growth was observed at 3 and 24 h compared to bacteria grown in the anaerobic cabinet; instead, a slight increase in *C. difficile* growth in the VDC was noted at 24 h (Supplementary Figure S2).

### *C. difficile* Colonization Leads to Disruption of the Intestinal Epithelium

To determine how *C. difficile* interacts with the human host in the short and long term, Caco-2/HT29-MTX layers were infected with *C. difficile* R20291 at a multiplicity of infection (MOI) of 100:1 for different periods of time in the anaerobic chamber of the VDC (Figure 1). In order to study bacteria that adhere to the IECs and their replication, for all experiments, at 3 h p.i., the apical supernatant containing the *C. difficile* was removed, the IECs washed in PBS, fresh prereduced media added, followed by incubation for the required time. Uninfected controls shown in Figure 2A were run in parallel. The number of adherent *C. difficile* (vegetative cells) was determined by counting colony forming units (CFUs) from the cell lysates, after washing off non-adherent bacteria. A significant increase in the number of cell-associated *C. difficile* was observed from 3 to 24 h p.i. (Figure 2C). This increase in cell-associated bacteria corresponded to a decrease in TEER measurements [from 100% (578 ± 141 Ω) at 3 h to 30.78% (181 ± 39.9 Ω) at 48 h] indicating disruption of the intestinal epithelial barrier (Figure 2B and Supplementary Figure S3A). Confocal microscopy showed *C. difficile* present as small micro-communities on the IECs at 24 and 48 h (Figure 2D). An additional image of micro-communities formed at 24 h is shown in Supplementary Figure S3B. At early time points (3 and 6 h p.i.), there was little disruption of the actin filaments but at 24 and 48 h p.i., destruction of the cytoskeleton was evident (Figure 2D). Interestingly, at 48 h p.i., immunofluorescent staining of bacteria showed the presence of filamenting *C. difficile* (Figure 2D). A Coloc 2 ImageJ colocalization analysis revealed partial colocalization of *C. difficile* with actin (Figure 2D) as indicated by the Manders’ M2 value (channel 2 for *C. difficile*) which shows 30 and 20% *C. difficile* colocalized with the actin at 24 and 48 h p.i., respectively (Manders et al., 1993; Table 1). Positive Li’s ICQ values and Costes signifance test values (Table 1) further confirmed colocalization at both time points (Costes et al., 2004; Li et al., 2004).

**TABLE 1:** Colocalization analysis using ImageJ. Coloc 2 was used to determine the colocalization of *C. difficile* with actin.

Coloc tests	24 h	48 h
Li ICQ	0.135 ± 0.0768	0.19 ± 0.0476
Manders tM1	0.16675 ± 0.1804	0.399 ± 0.0588
Manders tM2	0.3012 ± 0.2089	0.2087 ± 0.1040
Costes *P*-value	1	1

*Note: tM1 and tM2 represent the thresholded values for Manders’ coefficient, M1 and M2. The maximum value that can be obtained for Manders and Costes is 1 and for Li ICQ, 0.5 (ICQ, intensity correlation coefficient). The Costes *P*-value if >95% or 0.95 was deemed to be significant, so a *P*-value of 1 means that colocalization of *C. difficile* with actin was significant at 24 and 48 h. For the Li ICQ, the values range from maximum 0.5 to −0.5, i.e., with random (or mixed) staining ICQ = ∼0; dependent staining 0 < ICQ ≤ +0.5, and for segregated staining 0 > ICQ ≥ −0.5 (Li et al., 2004). Positive Li ICQ values also indicated dependent staining of *C. difficile* colocalizing with actin.*

### Prolonged Infection Is Associated With Spore and Toxin Production, and Host Responses

**FIGURE 3:**
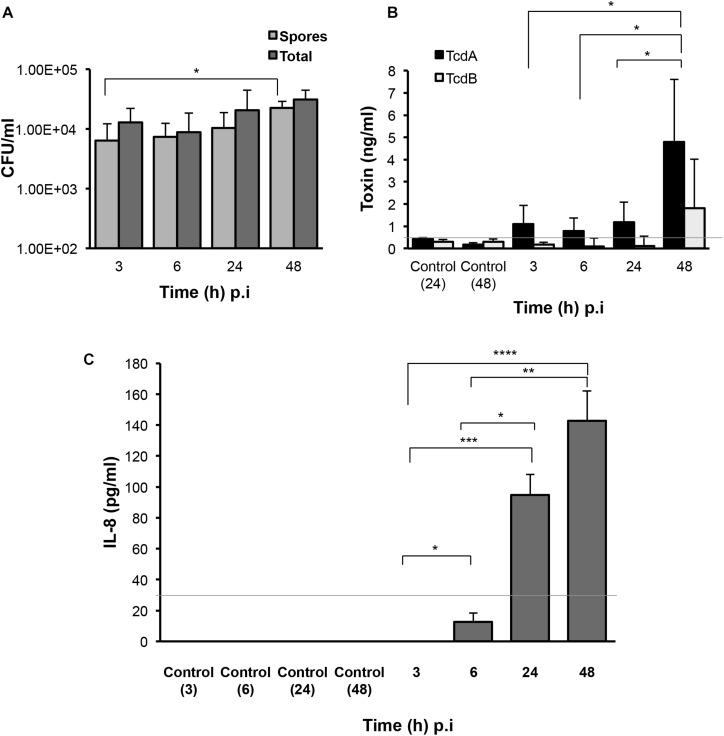
Production of *C. difficile* spores, toxins, and host responses to infection. **(A)** Colony counts of spores recovered after heat treatment, and total cells in the cell-associated *C. difficile* fraction (infected cell lysates) in the 2D epithelial model. Data shown are the mean of three independent experiments and error bars indicate SD, *p < 0.05 as determined by two-way ANOVA. **(B)** ELISA for *C. difficile* toxins A and B shows increased toxin production after extended infection. Toxins were measured from medium obtained from the apical compartment containing uninfected cell layers incubated for 24 or 48 h (Control) or cells infected with *C. difficile* for 3, 6, 24, or 48 h. Data shown are the mean of three independent experiments and error bars indicate SD, *p < 0.05 as determined by two-way ANOVA. Gray line represents the sensitivity of the test at 0.5 ng/ml. **(C)** ELISA for human IL-8 indicates increased IL-8 production at 24 and 48 h p.i. IL-8 was measured in medium obtained from the basolateral compartment containing uninfected cell layers incubated for 3, 6, 24, or 48 h (Control) or cells infected with *C. difficile* for 3–48 h. Gray line represents the limit of detection at 32 pg/ml. Data shown are the mean of three independent experiments and error bars indicate SD, *p < 0.05, **p < 0.01, and ***p < 0.001, ****p < 0.0001 as determined by one-way ANOVA with Tukey’s test for multiple comparison.

In order to fully understand bacterial factors necessary for CDI persistence, we studied the production of spores, toxins and a host chemokine in the E-VDC model. We measured spores and total bacteria from the cell-associated fraction at different times after infection. Although there were ∼0.1% spores in the inoculum, there was a mild but significant increase in spore numbers from 3 to 48 h p.i. (*p* < 0.05, Figure 3A). After washing off the non-adherent bacteria at 3 h, we also tracked the bacterial spore numbers from 3 to 48 h in the supernatants (Supplementary Figure S4). By 48 h p.i., there were equal numbers of spores and total cells in the supernatants (Supplementary Figure S4).

Toxins A and B (TcdA and TcdB) were monitored over time during infection using ELISA. Toxin A increased significantly from 3 to 48 h p.i. (*p* < 0.05) while low levels of toxin B were detected at all times (Figure 3B). Analysis of the basolateral compartment supernatant for the host chemokine IL-8, which has been previously implicated in CDI (Rao et al., 2014), revealed that IL-8 levels were low at 6 h p.i. but increased at 24 and 48 h p.i. in this infection model compared to uninfected controls (Figure 3C).

### Co-culturing *C. difficile* With *Bacteroides dorei* Results in Reduced *C. difficile* Growth Within an Epithelial Gut Model

**FIGURE 4:**
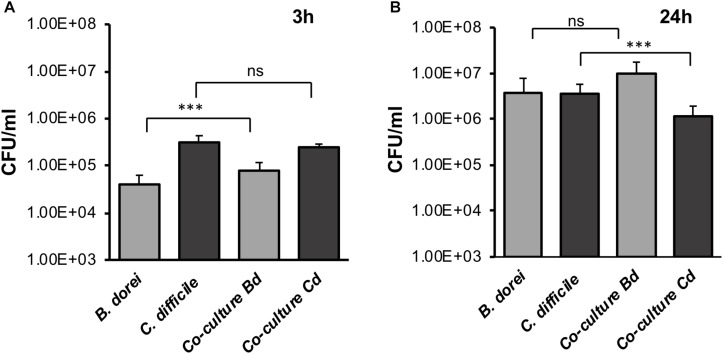
Studying *C. difficile*-commensal interactions within the E-VDC model. **(A)** Mono or mixed cultures of *B. dorei* and *C. difficile* were incubated with the epithelial monolayers in the VDCs for 3 h. Co-cultures of *B. dorei* with *C. difficile* in the VDC showed no significant reduction of vegetative *C. difficile* growth at 3 h p.i. as compared to *C. difficile* mono-culture. A relative increase in *B. dorei* adhering to the IECs was observed in co-cultures. “Co-culture Bd and Cd” represent the growth of *B. dorei* and *C. difficile*, respectively, in the mixed culture (Bd, *B. dorei* and Cd, *C. difficile*) on the IECs. Data shown are the mean of three independent experiments and error bars indicate SD, ***p < 0.001, ns, not significant as determined by one-way ANOVA. **(B)** At 24 h, a significant reduction of *C. difficile* CFU (vegetative cells) is observed in co-cultures compared with *C. difficile* mono-culture. Data shown are the mean of three independent experiments and error bars indicate SD, ***p < 0.001, ns, not significant as determined by one-way ANOVA with Tukey’s test for multiple comparison.

To determine if our E-VDC system could be extended for use with other anaerobic bacteria, we studied a strict commensal anaerobe, *B. dorei* within the E-VDC model using similar culture conditions as for single CDIs, except that a higher MOI (500–1000:1) was used. Adherence of vegetative bacterial cells to the IECs was determined at 3 and 24 h by CFU counts, as described for single species infection. *B. dorei* adhered to the epithelial cells at 3 h and multiplied over 24 h, as observed for *C. difficile* (Figures 4A,B). A mixed culture of *B. dorei* and *C. difficile* (1:1), prepared as described in Section “Materials and Methods” was then cultured with the monolayer in the VDC. Cell-associated bacteria were quantitated by plating on a medium used to isolate *C. difficile* colonies, which also allowed the growth of *B. dorei*. *Bacteroides* colonies were distinguished by colony size, color, and morphology (small colonies). A significant decrease in the number of *C. difficile* was observed when co-cultured with *B. dorei* in the presence of IECs when compared to mono-cultures of *C. difficile* at 24 h p.i., but not at 3 h p.i. (Figures 4A,B, *p* < 0.001). We also observed higher colony counts when *B. dorei* was grown in co-culture with *C. difficile* at 3 and 24 h, compared to mono-cultures of *B. dorei* (Figures 4A,B).

### Development of Complex *C. difficile* Infection Models

**FIGURE 5:**
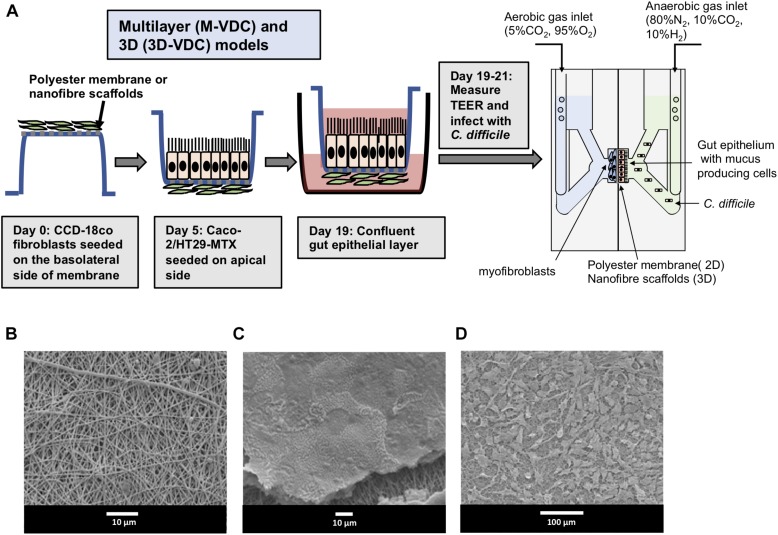
Scheme for generation of multilayer (M-VDC) and 3D (3D-VDC) models containing fibroblast cells and cell growth on the electrospun nanofibrous matrix. **(A)** Fibroblast cells were first seeded on the basolateral side of the Snapwell or nanofiber scaffold for the M-VDC and 3D-VDC models for 5 days. Thereafter, IECs were cultured on the apical side for 2 weeks before placing the Snapwell insert between two halves of the chamber. *C. difficile* was infected apically while maintaining anaerobic conditions and the basolateral compartment was perfused with 5% CO_2_ and 95% air. **(B)** Polyethylene terephthalate (PET; 10% w/v) was electrospun and exhibited a uniform nanofibrous matrix as determined by scanning electron microscopy. **(C)** Caco-2 epithelial cells were able to form a confluent monolayer on the nanofibrous scaffolds (the scaffold can be seen underneath the cells following processing for SEM). **(D)** The CCD-18co cells proliferated across the nanofibrous scaffold forming cell processes to adhere to the nanofibers.

Typically, a myofibroblast layer underlies the basement membrane in the human gut. To develop this system further by increasing its cell complexity, we incorporated myofibroblasts into our model. Human CCD-18co myofibroblasts were first grown on the basolateral side of the polyester Snapwell insert before seeding the IECs on the apical side [multilayer VDC (M-VDC) model, Figure 5A]. These inserts were then placed in the VDC and infections were performed as described in Materials and Methods.

Furthermore, to recreate the highly porous architecture of the ECM in the basement membrane, epithelial and fibroblasts cells were grown apically and basolaterally, respectively, on inserts containing electrospun nanofiber scaffolds generated from PET (3D-VDC). The electrospun scaffolds provide optimal 3D topography for cell growth as reported previously (Morris et al., 2014). The scaffolds were then inserted into the VDC and the infections performed. Scanning electron microscopy revealed that these scaffolds exhibited a uniform nanofibrous matrix (Figure 5B) that supported the attachment and proliferation of Caco-2 cells to form a confluent monolayer (Figure 5C) and enabled the proliferation of the CCD-18co cells (Figure 5D). The average fiber diameter was 457 ± 170 nm (Supplementary Figure S5).

### Increased Cell-Associated Bacteria in the M-VDC and 3D-VDC Models

**FIGURE 6:**
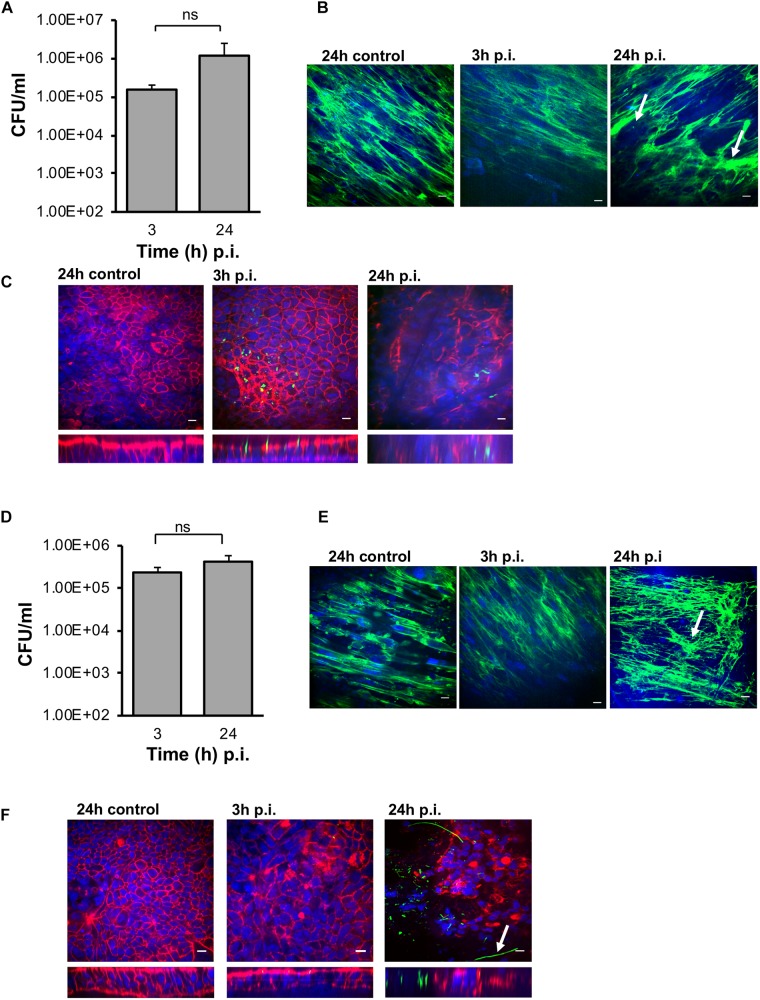
Infection of *C. difficile* in M-VDC and 3D-VDC model leads to increased adhesion. Increased adherence of *C. difficile* in the M-VDC and 3D-VDC models, where fibroblasts are grown as a basolateral layer on polyester and nanofibers, respectively. **(A)** Serial dilutions of the infected epithelial cell lysates were plated to determine the number of cell-associated bacteria (vegetative cells) in the M-VDC model. Data shown are the mean of three independent experiments and error bars indicate SD, ns, not significant as determined by student’s *t*-test. **(B)** Anti-fibronectin (green) staining of fibroblast cells in the basolateral layer of the control at 24 h p.i and infected cells at 3 and 24 h p.i. showing redistribution of fibronectin, damaged fibroblast layer in the M-VDC model, as indicated by a white arrow. **(C)** Representative images of *C. difficile* on the apical layer at 3 and 24 h p.i. Inset below shows the orthogonal *XZ* axis view of the IECs. *C. difficile* is stained green, actin, red, and cell nuclei, blue, scale bar = 10 μM. **(D)** *C. difficile* colony counts (vegetative cells) from the 3D model infected cell lysates. Data shown are the mean of three independent experiments and error bars indicate SD, ns, not significant as determined by student’s *t*-test. **(E)** Anti-fibronectin (green) staining of fibroblast cells in the basolateral layer at 3 and 24 h p.i. **(F)** Immunofluorescent microscopy images of *C. difficile* infected IECs at 3 and 24 h p.i. in the 3D model. *C. difficile* filamentous forms are observed at 24 h p.i. (indicated by arrow). Inset below shows the orthogonal *XZ* axis view of the IECs. *C. difficile* is stained green, actin, red, and cell nuclei, blue, scale bar = 10 μM.

Interestingly, in the M-VDC model, after infection with the same *C. difficile* strain, same MOI’s (100:1) and conditions as the E-VDC model, for 3 and 24 h (Figure 2), higher numbers of vegetative bacterial cells adhered to the IECs at both time points (Figure 6A) compared to the E-VDC model without fibroblasts (Figure 2). Anti-fibronectin staining of the basolateral side of the membrane containing the fibroblast cells indicated likely degradation of fibronectin, as indicated by the destabilized fibronectin network, and damage to the fibroblast layer at 24 h p.i. (Figure 6B). As before, immunofluorescent staining showed the localization of *C. difficile* on the epithelial cells at 3 and 24 h p.i. (Figure 6C). Infections were not followed for longer times (48 h) as the cell layer was badly damaged by 24 h p.i.

Similar to the M-VDC model, we observed higher adhesion of *C. difficile* (vegetative cells) to the IECs at 3 and 24 h p.i. in the gut epithelium in the 3D-VDC model (Figure 6D) compared to the E-VDC model data in Figure 2. Similarly, anti-3D fibronectin staining showed a destabilization of the discrete fibronectin network and likely cellular damage of the fibroblast layer in the 3D-VDC model, as indicated by the lack of discrete nuclear staining (Figure 6E). Confocal microscopy revealed the presence of numerous *C. difficile* on the IECs at 24 h p.i. (Figure 6F). Interestingly, at 24 h p.i., immunofluorescent staining showed the presence of filamenting *C. difficile* (Figure 6F), much earlier than seen in the E-VDC infection model. Bacterial staining did not always correlate to the CFU counts (Figures 6C,F), which we attribute to the loss of attached bacteria during the staining procedure.

### Spore, Toxin, and Host Chemokine Production in Response to *C. difficile* in M-VDC and 3D-VDC Models

**FIGURE 7:**
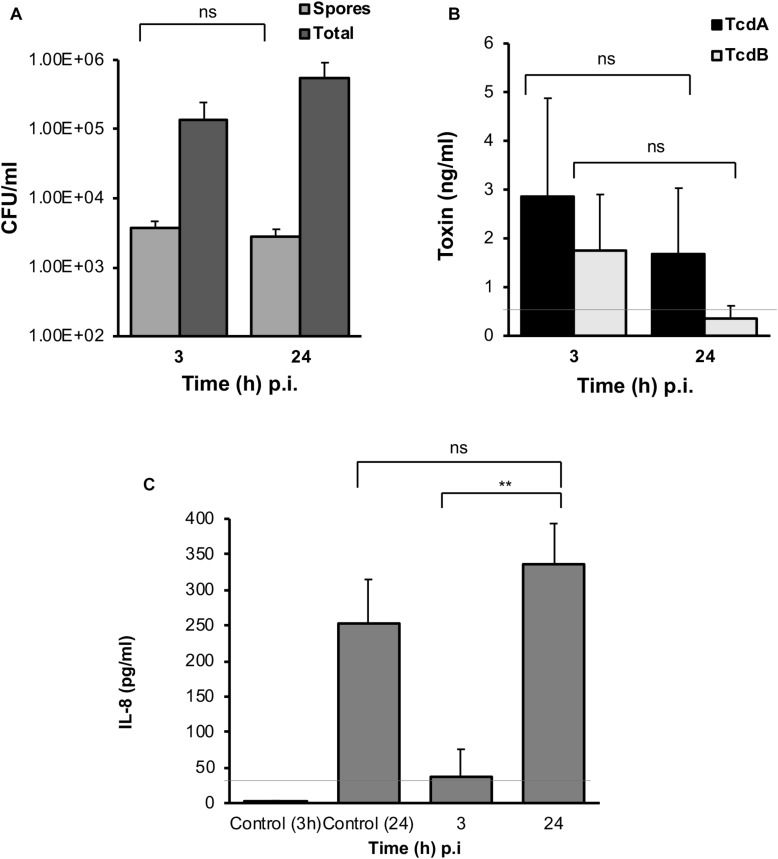
*C. difficile* spores and toxin production, and host response to infection in the 3D-VDC model. **(A)** Colony counts of spores and total cells in the host cell-associated *C. difficile* fraction (infected cell lysates). Data shown are the mean of three independent experiments and error bars indicate SD, ns, not significant as determined by two-way ANOVA. **(B)** Toxin A and B levels from apical compartment supernatants as determined by ELISA in the 3D model. Data shown are the mean of three independent experiments and error bars indicate SD, ns, not significant as determined by two-way ANOVA. Gray line represents the sensitivity of the test at 0.5 ng/ml. **(C)** Human IL-8 levels in supernatants from the basolateral compartments with uninfected cells incubated for 24 h or with cells infected with *C. difficile* for 3 and 24 h, as determined by ELISA. Gray line represents the limit of detection at 32 pg/ml. Data shown are the mean of three independent experiments and error bars indicate SD, **p < 0.01, as determined by the one-way ANOVA with Tukey’s test for multiple comparison.

In both the M-VDC and 3D-VDC models of infection, spores were found to adhere to the epithelium at numbers comparable to the E-VDC model (Figures 3A, 7A and Supplementary Figure S6A), although there was no increase in spore numbers over time. A higher increase in total cell numbers was observed over time in the M-VDC and 3D-VDC models (Figure 7 and Supplementary Figure S6A), unlike the E-VDC model (Figure 3A). While there was higher variability, levels of toxin A in the apical compartment supernatants from both models were comparable to that produced in the E-VDC models (Figure 7B and Supplementary Figure S6B). Levels of toxin B detected in these models were also low (Figure 7B and Supplementary Figure S6B). Levels of IL-8, increased over time (3–24 h p.i.) in the M-VDC (Supplementary Figure S6C) and 3D-VDC models (Figure 7C), although these were not significantly different to the uninfected controls incubated for 3 or 24 h within the VDCs, as there was an unexpected increase in IL-8 produced by the control cells at 24 h.

## Discussion

Attachment to the intestinal mucosa, subsequent multiplication, and penetration of the gut epithelium are generally essential for the establishment of a successful invasive infection (Kalita et al., 2014; Ribet and Cossart, 2015). We report here the development of new cellular human gut models for studying interactions of obligate anaerobes with the host gut epithelium. We have tracked CDI events over a prolonged time frame on a monolayer epithelium (E-VDC) or in complex models comprising of epithelial and myofibroblast layers (M-VDC, 3D-VDC). Along with demonstrating *C. difficile* adhesion, micro-communities, toxin, and spore production, we report formation of *C. difficile* filaments, a potential adaptation mechanism during infection.

Previous cellular infection models have shown the attachment of *C. difficile* on IECs either using conditions that are not appropriate for the bacterium (such as growing in aerobic conditions) or that are limited to short term infection due to the anaerobic growth requirements of *C. difficile* (Cerquetti et al., 2002; Paredes-Sabja and Sarker, 2012; Jafari et al., 2016). However, in order to study host–pathogen interactions at a molecular and cellular level, precise environmental control and longer scales of infection are essential. The models described here are ideal for investigating molecular mechanisms underlying *C. difficile*–epithelial interactions as it offers the ability to make cellular, immunological, and molecular measurements along with easy substitution of knockout cell lines and introduction of additional cellular components like immune cells. CDI has been studied previously within the VDC with a T84 cell layer (Jafari et al., 2016). In this study, the authors compared *C. difficile*-induced cytokine production and epithelial barrier disruption between aerobic and anaerobic conditions. In our study, we have tried to better represent the gut epithelium by using a mix of Caco-2 and mucus-producing HT29-MTX cells, and have followed infection, in particular, cell-associated bacteria, over a longer time frame.

In the E-VDC model, an increase in spores attached to the host cells was observed at 48 h p.i. indicating that spore formation occurs during CDI of the gut epithelium in our monolayer model, as reported previously using mouse models (Deakin et al., 2012). While we cannot be sure if spores are formed and then adhere or if the vegetative cells adhere to the gut cells and sporulate, our findings clearly support previous studies indicating spore adhesion to gut cells during infection. *C. difficile* spores were reported to adhere to undifferentiated Caco-2 cells after 1 h of infection as determined by viable spore counts and fluorescence microscopy (Paredes-Sabja and Sarker, 2012), and recently, a spore surface protein CotE was shown to be essential for spore binding to mucus producing epithelial cell layers (Hong et al., 2017).

Toxin production by *C. difficile* is known to play a role in pathogenesis by disrupting the barrier integrity of the intestinal epithelium leading to increased permeability and re-organization of actin (Aktories et al., 2017). Surprisingly, although there is a decrease in TEER and actin reorganization, we detect very low levels of toxins at early time points of infection (6–24 h). It is possible that low toxin levels, particularly of toxin B, are sufficient to cause loss of the membrane integrity, as they are augmented by other secreted enzymes produced by *C. difficile*. Although a partial bacterial colocalization with actin was observed later during infection (24 and 48 h), we do not at present understand if *C. difficile* mediates any direct interactions with the actin cytoskeleton. The formation of *C. difficile* filaments seen at 24 and 48 h p.i. may be associated with host cell contact and infection, as our studies indicate that incubation in similar conditions (conditioned medium from infected cells or growth in DMEM-10 for 48 h) in the absence of cells does not induce this phenotype (Supplementary Figures S7A,B). *C. difficile* filaments may be important during infection, as seen for other gut bacteria (Justice et al., 2008), although this needs to be investigated further.

More than 90% of the bacteria inhabiting the gut are obligate anaerobes but little is known about the adhesion properties of individual bacteria to the intestinal epithelium. Previous research on *Bacteroides fragilis*, a dominant bacterium isolated from intestinal tract infections showed that bile salts enhance the adhesion to the intestinal epithelium and biofilm formation (Pumbwe et al., 2007). However, for other dominant members of the Bacteroidetes phylum, there is insufficient data as to how they interact with the gut epithelium. Our studies with *B. dorei*, a common gut anaerobe in healthy individuals, demonstrates bacterial adhesion and multiplication on IECs, and validates the utility of this model in studying host cell interactions of other anaerobic gut bacteria.

While the gut microbiota is known to protect against CDI (Fuentes et al., 2014), the direct interactions between commensals and gut pathogens have been poorly understood. Although many genome sequencing studies have associated members of the Bacteroidetes with healthy status, the roles of individual *Bacteroides* species in the gut remain unclear (Qin et al., 2010; Huttenhower et al., 2012; Lloyd-Price et al., 2016). Recently, it was shown that *Bacteroides ovatus* inhibited *C. difficile* growth in the presence of bile acids (Yoon et al., 2017). In this study, we demonstrate suppression of gut cell-associated *C. difficile* growth by *B. dorei*, in the absence of bile acids and in a physiologically relevant setting. Indeed, the reduction in *C. difficile* may be because *B. dorei* competes better for nutrients, rather than a direct inhibition. In addition to host–pathogen interactions, the models developed in this work allow three-way interaction studies between commensals, pathogens, and the host, i.e., the role of commensals in modulating effects of pathogenic bacteria on the human host. Additionally, as these results may also suggest the potential of non-spore forming anaerobic bacteria in suppressing *C. difficile* growth, such *in vitro* tools may have value in screening and developing novel microbial therapies for treatment of *C. difficile* and other anaerobic infections.

Three-dimensional models are known to promote more *in vivo*-like cell proliferation, growth differentiation, and cell-to cell contact (Kook et al., 2017). A recent study reported a 3D intestinal tissue model for *C. difficile*, where the authors demonstrated that *C. difficile* toxin activity was higher in comparison to a 2D transwell model (Shaban et al., 2018). They report that spores can germinate into vegetative cells within this model and that vegetative cells can survive up to 48 h, although the ability of *C. difficile* spores to germinate was similar in both their models. While both their 3D model and our models have epithelial and fibroblast cells, albeit with different scaffolds, a key difference is the ability to control the apical and basolateral side environments using the VDC, which may be important when performing sensitive molecular studies. Similar to Shaban et al. (2018), we show bacterial replication up to 48 h, although we also track bacteria that adhere to the epithelial layer. Additionally, we have a nanofiber scaffold incorporated between the epithelial and fibroflast layers, which creates a porous architecture similar to the basement membrane underlying the gut epithelium, along with myofibroblast cells in the basal layer.

Previously, Morris et al. (2014) showed that nanofibers were beneficial for epithelial cell differentiation but not penetrable to fibroblasts. In addition to producing an ECM which provides structural and biochemical support to cells, fibroblasts are also known to produce chemokines when activated by bacteria (Smith et al., 1997). The increased *C. difficile* adhesion observed in the multilayer (M-VDC) and the 3D (3D-VDC) models, which presumably leads to quicker progressing infection, could be the result of indirect modulation of the epithelial barrier by the myofibroblast cells, though at present we do not understand the underlying mechanisms. The increased amount of the chemokine IL-8 observed in the uninfected 3D and M-VDC models could be attributed to the myofibroblasts, although this chemokine response is not specific to CDI. It is interesting to note that inspite of having higher numbers of bacteria attaching in these models, there was no significant increase in the toxin levels. While we do not observe an increase in spore numbers or distinct toxin production profiles in the 3D-VDC and M-VDC models, this may be due to the quick progression of infection due to higher bacterial adhesion. We are currently optimizing these models using lower MOIs to enable longer infection experiments.

Both the multilayer and 3D gut models have huge potential in studying *C. difficile* pathogenesis, particularly for investigating host interactions with basolateral surface of the host epithelium and are being utilized for studying functions of secreted *C. difficile* factors in the laboratory. Overall, we have developed highly useful tools for studying *C. difficile*–host interactions which we expect to have broad applications in studying anaerobic gut commensals and their interactions with pathogens and the host.

## Materials and Methods

### Bacterial Strains and Growth Conditions

*Clostridium difficile* R20291 strain B1/NAP1/027 R20291 (isolated from the Stoke Mandeville outbreak in 2004 and 2005), was cultured on brain–heart infusion (BHI) agar (Sigma-Aldrich, United Kingdom) under anaerobic conditions (80% N_2_, 10% CO_2_, 10% H_2_) in a Don Whitley workstation (Yorkshire, United Kingdom).

### Cell Culture, Media, and Conditions

Intestinal epithelial cell line, Caco-2 (P6-P21) from American Type Culture Collection, mucus producing cell line, HT29-MTX (P45-P56), gift from Nathalie Juge, Quadram Institute, Norwich, and fibroblast cells, CCD-18co (P10-P17) were used. Caco-2 cells were grown in DMEM supplemented with 10% FBS (Labtech, United Kingdom), and 1% penicillin-streptomycin (10,000 units/mL penicillin, 10 mg/mL streptomycin, Sigma-Aldrich, United Kingdom). HT29-MTX was grown in DMEM and CCD-18co in Eagle’s Minimum Essential Medium media. Both media were supplemented with 10% FBS, 1% penicillin-streptomycin, 2 mM glutamine, and 1% non-essential amino acids (Sigma-Aldrich, United Kingdom). All cell lines were maintained in 5% CO_2_ in a humidified incubator at 37°C and free from mycoplasma contamination as determined by the EZ-PCR Mycoplasma kit (Biological Industries, United States).

For the epithelial 2D models, Caco-2 and HT29-MTX were mixed in a 9:1 ratio and 2 × 10^5^ cells/ml were seeded on a 12 mm Snapwell inserts (tissue culture treated polyester membrane, Corning, New York, NY, United States) supported by a detachable ring for 2 weeks to form a polarized monolayer. Prior to seeding the cells, the snapwell inserts and nanofiber scaffolds were coated with a 1:1 ratio of rat tail collagen (Sigma-Aldrich, United Kingdom) and ethanol and allowed to dry. For the multilayer (M-VDC) and 3D (3D-VDC) models, CCD-18co (5 × 10^4^ cells/ml) were first seeded on the basolateral layer of the polyester Snapwell insert or electrospun nanofiber scaffold for 5 days after which Caco-2 and HT29-MTX were seeded on the apical side as in the 2D models for 14 days. Prior to the infection experiments, the cell culture medium in the Snapwell inserts was replaced with antibiotic-free medium.

### Vertical Diffusion Chamber Setup and Measurement of Transepithelial Electrical Resistance

The Snapwell inserts containing the polarized cell layer were placed between the two half chambers of the VDC (Harvard Apparatus, Cambridge, United Kingdom) and sealed with the provided clamps; 2.7 ml DMEM with 10% FBS (DMEM-10) was placed on both sides of the chamber. TEER measurements were performed using Harvard/Navicyte electrodes on the EC-800 Epithelial Voltage Clamp (Harvard Apparatus, Cambridge, United Kingdom) over 24 h.

### Infection of Intestinal Epithelial Cells (IECs) in E-VDC, M-VDC, and 3D-VDC Models

A single bacterial colony was inoculated in prereduced BHI broth (Oxoid, United Kingdom) supplemented with 1 g/L cysteine (Sigma-Aldrich, United Kingdom) and 5 g/L yeast extract and incubated at 37°C overnight. The culture was centrifuged at 10,000 *g* for 5 min (Eppendorf 5810R) and bacterial pellet was resuspended in DMEM-10 and incubated at 37°C for at least an hour. Bacterial counts determined from this culture were confirmed to be 2 × 10^7^–3 × 10^7^ CFU/ml for every experiment. This culture was used to infect the IECs at a predetermined MOI of 100:1 in the apical side of the VDC containing the IECs. The apical chamber was diffused with anaerobic gas mixture (10% CO_2_, 10% H_2_, 80% N_2_, BOC, United Kingdom) and the basolateral compartment with 5% CO_2_ and 95% air (BOC, United Kingdom). At 3 h p.i., the apical media containing the *C. difficile* was removed, the IECs washed in PBS and 2.7 ml prereduced DMEM with 10% FBS added. It was incubated for a further 3 or up to 48 h. The apical and basolateral media was then removed and stored at −80°C. The IECs were washed thrice in prereduced PBS before lysing in 1 ml sterile water. Serial dilutions prepared from the IEC lysates were performed and plated on BHI agar to determine the number of cell-associated bacteria.

### Spore and Total Cell Counts

To determine the number of spores, the lyzed cells and apical supernatants were heat treated at 65°C for 20 min as previously described (Fimlaid et al., 2015). Untreated and heat treated samples were serially diluted and plated on BHI and BHI-T agar (supplemented with 0.1% sodium taurocholate, Sigma-Aldrich, United Kingdom). No bacteria were obtained from the heat-treated samples plated on BHI (without sodium taurocholate). The CFU/ml obtained from heat treated samples plated on BHI-T plates represent heat-resistant spores, and the CFU/ml obtained from untreated samples plated on BHI-T plates represent the total cell counts.

### Co-culture Experiments

For the co-culture experiments with *B. dorei*, both strains were grown to stationary phase overnight in Schaedler anaerobic broth (Oxoid, United Kingdom), centrifuged with pellets resuspended in DMEM-10. OD_600_ of the suspensions were measured but not used to normalize cultures, as we found that for *B. dorei* the OD_600_ does not correlate well with CFU/ml. Both cultures were diluted 1:1 in DMEM-10 before loading into the VDC. The CFU/ml of these diluted cultures were determined to ensure that equal numbers of *C. difficile* to *B. dorei* (5 × 10^7^–2.9 × 10^8^ CFU/ml) were present. Equal volumes of the diluted cultures were mixed prior to loading into the VDC. An MOI of 500–1000:1 was used for both bacterial species. To differentiate *C. difficile* colonies from *B. dorei*, BHI agar was supplemented with *C. difficile* selective supplement containing D-cycloserine and cefoxitin (Oxoid, United Kingdom).

### ELISA Assays

*Clostridium difficile* toxin production was determined using the *C. difficile* toxin A or B kit by ELISA following the manufacturer’s instructions (tgcBIOMICS, Bingen am Rhein, Germany). Briefly, the apical supernatants were centrifuged at 2500 *g* for 5 min and 100 μl of the supernatant in duplicates was used for the assay. Duplicates (100 μl) of the standards (toxin A and B) provided with the kit was run in the same ELISA assay as the apical supernatants from which the amount of toxin produced was calculated. IL-8 production was also determined by analysis of basolateral supernatants from the VDC using a human IL-8 ELISA kit (R&D systems, Minneapolis, MN, United States) following the manufacturer’s instruction. Duplicates (100 μl) of the standards (IL-8) provided with the kit was run in the same ELISA assay as the basolateral supernatants from which the amount of IL-8 produced was calculated.

### Electrospinning Protocol

Electrospinning procedure was performed as described previously (Morris et al., 2014). Briefly, scaffolds were produced by dissolving PET (from food grade drinking bottles) in equal ratio of trifluoroacetic acid and dichloromethane (1:1) to create a 10% (w/v) PET solution to produce the nanofiber scaffolds. Electrospinning processing parameters included an applied voltage of 14 kV, a tip-collector distance (TCD) of 15 cm, a flow rate of 0.5 ml/h, and an 18 gage spinneret for a period of 2 h. Electrospinning was conducted at ambient conditions in a ducted fume hood with fibers collected on a stainless steel rotating drum (50 rpm). Once collected, the scaffolds were stored in aluminum foil at room temperature until required. Prior to cell culture, scaffolds were positioned in 12 mm Snapwell inserts (Corning, New York, NY, United States) by replacing the commercial membrane with the electrospun nanofiber matrix. PET scaffolds were cut into 2.5 cm diameter circles and fixed with Costar Snapwell inserts using aquarium sealant glue (King British, Beaphar Company). They were sterilized by exposing to UV at the power of 80 mJ/cm^2^ for 15 min on each side using a UV lamp (CL-1000 Ultraviolet Crosslinker) and soaking in 10x antibiotic-antimycotic solution (final concentration: 1000 units/ml penicillin, 1 mg/ml streptomycin, and 2.5 μg/ml amphotericin B, Sigma-Aldrich, United Kingdom) for 3 h at 25°C. Antibiotic-antimycotic solution was then removed and the scaffolds were washed with PBS (pH 7.4) three times. The scaffolds were air-dried in the microbiological class 2 safety cabinet overnight.

### Scanning Electron Microscopy (SEM)

The fiber morphology of the nanofiber scaffolds was assessed using SEM. Acellular samples were sputter coated (Leica EMSCD005) with gold for 4–5 min prior to microscopy. Scaffolds seeded with cells were fixed in 3% (v/v) glutaraldehyde overnight at 4°C. Samples were washed thrice in PBS and dehydrated through a series of industrial methylated spirits (IMS) concentrations diluted in water (25–100% v/v) for 10 min each. Following dehydration, hexamethyldisilazane (HMDS) was added to chemically dry the samples, this process was then repeated and the samples allowed to air dry overnight before sputter coating as described above. Scaffold and cell morphology was observed at various magnifications as indicated on the scale bar of the images (JEOL JSM-6100, JEOL, United Kingdom). Images were processed using ImageJ software (W. Rasband, National Institute of Health, United States) to determine the fiber diameter and 150 measurements (50 measurements per scaffold) from three independently produced scaffolds were assessed.

### Immunofluorescence Assays and Imaging

The Snapwell inserts, containing either the commercial membrane or PET nanofiber scaffold, were fixed in 4% PFA for 15 min, washed in PBS, permeabilized with 1% saponin (Sigma-Aldrich, United Kingdom) in 0.3% triton X-100 (Fisher Scientific, United Kingdom) and thereafter blocked against non-specific antibody binding with 3% BSA in PBS (Sigma-Aldrich, United Kingdom). For staining *C. difficile*, infected cells were incubated with anti-*C. difficile* serum for 1 h, followed by Alexa Fluor 488 goat anti-rabbit secondary antibody (New England Biolabs, United Kingdom) for 1 h. Alexa fluor phalloidin 647 was used to stain the actin cytoskeleton and cell nuclei were stained with ProLong Gold Antifade mountant containing 4,6-diamidino-2-phenylindole (DAPI) (New England Biolabs, United Kingdom). To determine mucus production, the Snapwell inserts were fixed, permeabilized, and blocked as described above. The Snapwell insert was incubated with mucin 2 anti-rabbit primary antibody (Santa Cruz Biotechnology, United States) overnight at 4°C followed by Alexa Fluor 488 goat anti-rabbit secondary antibody (New England Biolabs, United Kingdom) for 1 h. Images were taken using a confocal spinning-disk microscope (VOX UltraView, PerkinElmer, United Kingdom) with a 40X oil objective and two Hamamatsu ORCA-R2 cameras, by Volocity 6.0 (PerkinElmer, United Kingdom). Post-image analysis was performed with ImageJ software.

### Colocalization Analysis

Coloc 2 from the ImageJ software was used to determine the colocalization between *C. difficile*, visualized in green and actin in red, from four independent images at 24 and 48 h p.i. Coloc 2 runs several intensity based colocalization tests such as Manders correlation (Manders et al., 1993), Li intensity correlation quotient (ICQ) (Li et al., 2004), and Costes significance test (Costes et al., 2004). Intensity-based analysis such as Manders’ showed the level of colocalization between the two channels (channel 1 for actin and 2 for *C. difficile*). Manders’ output two results, M1 and M2 that describe the colocalization coefficients of the two channels. Manders’ M1 value showed that an average of 50% actin colocalized with *C. difficile* and Manders’ M2 value indicated that 100% of the *C. difficile* colocalized with actin at 24 h p.i (values between 0.5 and 1). At 48 h p.i, Manders M1 and M2 values showed that 100% of the actin colocalized with *C. difficile*. After applying an automatic threshold determined by the software, the levels of colocalization was lower (Table 1). One hundred randomizations were used to determine the Costes significant *P*-value. The Costes *P*-value if >95% or 0.95 was deemed to be significant. For all analyses except the Li ICQ, 1 represents perfect colocalization with lesser values indicating the various degree of colocalization. For the Li ICQ, the values range from maximum 0.5 to −0.5, i.e., with random (or mixed) staining ICQ = ∼0; dependent staining 0 < ICQ ≤ +0.5, and for segregated staining 0 > ICQ ≥ −0.5 (Li et al., 2004).

### Statistical Analysis

One-way or two-way anova was used to compare two or more groups when there was one or more independent variables, respectively, with Tukey’s test for multiple comparison using Graphpad Prism. Student’s *t*-test (two tailed) was used to determine the significance between two groups. Significance is represented as *p < 0.05, **p < 0.01, ***p < 0.001, and ****p < 0.0001. Except stated otherwise, all the results presented are the average of three independent experiments performed in duplicates or triplicates. The error bars indicate the standard deviation (SD).

## Author Contributions

BA, JH, and JP performed the experiments for this study. UD and AA produced the nanofiber scaffolds. BA, MU, FR, and SS were involved in designing experiments in the study. BA and MU wrote the main manuscript. All authors reviewed the manuscript.

## Conflict of Interest Statement

The authors declare that the research was conducted in the absence of any commercial or financial relationships that could be construed as a potential conflict of interest.

## Supplementary Material

FIGURE S1 Characterization of mucus production in the Caco-2/HT29-MTX monolayer. Immunofluorescent microscopy images of Caco2/HT29-MTX monolayer (control, uninfected) stained with mucin 2 antibody showing mucus production (green) after 14 days of cell culture in the Snapwell insert. Actin is stained red and cell nuclei, blue. Scale bar = 10 μM.

FIGURE S2 The vertical diffusion chamber maintains anaerobic conditions. Overnight cultures of *C. difficile* were centrifuged and equal numbers of bacteria (3.6 × 10^7^ CFU/ml) were resuspended in DMEM-10 and incubated in the VDC or anaerobic cabinet for 3 and 24 h. Serial dilutions of the apical supernatant were plated on BHI agar to enumerate colony counts. Data shown are the mean of three independent experiments and error bars indicate SD, **p* < 0.05, ns, not significant by one-way ANOVA.

FIGURE S3 **(A)** Change in TEER during *C. difficile* infection. Reduction in TEER measurements at different times after infection. **p* < 0.05, ^∗∗^*p* < 0.01, and ^∗∗∗^ *p* < 0.001 as determined by one-way ANOVA with Tukey’s test for multiple comparison. **(B)** Immunofluorescent microscopy image of *C. difficile* infected IECs at 24 h p.i. showing the bacteria stained with anti *C. difficile* antibodies (green) and actin stained with phalloidin (red). Cell nuclei are stained with DAPI (blue). Arrow indicates *C. difficile* present as micro-communities on the IECs.

FIGURE S4 Spore counts from apical chamber culture supernatants from the E-VDC model. Colony counts of spores and total cells in the apical compartment supernatants. A significant decrease in total cell count was seen at 24 and 48 h p.i. but an increase in spores compared to the total bacterial numbers is observed. Data shown are the mean of three independent experiments and error bars indicate SD, **p* < 0.05 as determined by two-way ANOVA.

FIGURE S5 Fiber diameter of the electrospun nanofibrous matrix. Fiber diameter analysis revealed that the average fiber diameter was 457 ± 170 nm, with fibers in the range of 200–1100 nm (*n* = 150 measurements, 50 measurements per scaffold from three independently produced scaffolds).

FIGURE S6 *C. difficile* spores and toxin production, and host response to infection in the M-VDC model. **(A)** Colony counts of spores and total cells in the host cell-associated *C. difficile* fraction of the M-VDC model. Data shown are the mean of three independent experiments and error bars indicate SD, ns, not significant as determined by two-way ANOVA. **(B)** Toxin A and B levels in the M-VDC model. Data shown are the mean of three independent experiments and error bars indicate SD, ns, not significant as determined by two-way ANOVA. Dashed line represents the sensitivity of the test at 0.5 ng/ml. **(C)** Human IL-8 levels in the M-VDC model. Data shown are the mean of three independent experiments and error bars indicate SD, ns, not significant as determined by the one-way ANOVA with Tukey’s test for multiple comparison.

FIGURE S7 Filaments are not produced when *C. difficile* is grown for 48 h in culture in the absence of intestinal epithelial cells. **(A)** *C. difficile* was grown for 48 h using DMEM-10 and stained with anti-*C. difficile* for 1 h, followed by Alexa Fluor 488 goat anti-rabbit secondary antibody (green). **(B)** The basolateral supernatant (DMEM-10) from a 24 h infection in the VDC was used to grow *C. difficile* for 48 h to determine if cell released factors in the supernatant can induce filament formation. Staining with anti *C. difficile* for 1 h was performed, followed by Alexa Fluor 488 goat anti-rabbit secondary antibody (green). Images were taken with a Leica DMi microscope at 100x magnification. Scalebar = 10 μM.
