# Supplementary figures and images for: Probing Clostridium difficile Infection in Complex Human Gut Cellular Models
Source: Front Microbiol. 2019 Apr 30;10:879. doi: 10.3389/fmicb.2019.00879 (PMC6503005; doi:10.3389/fmicb.2019.00879)

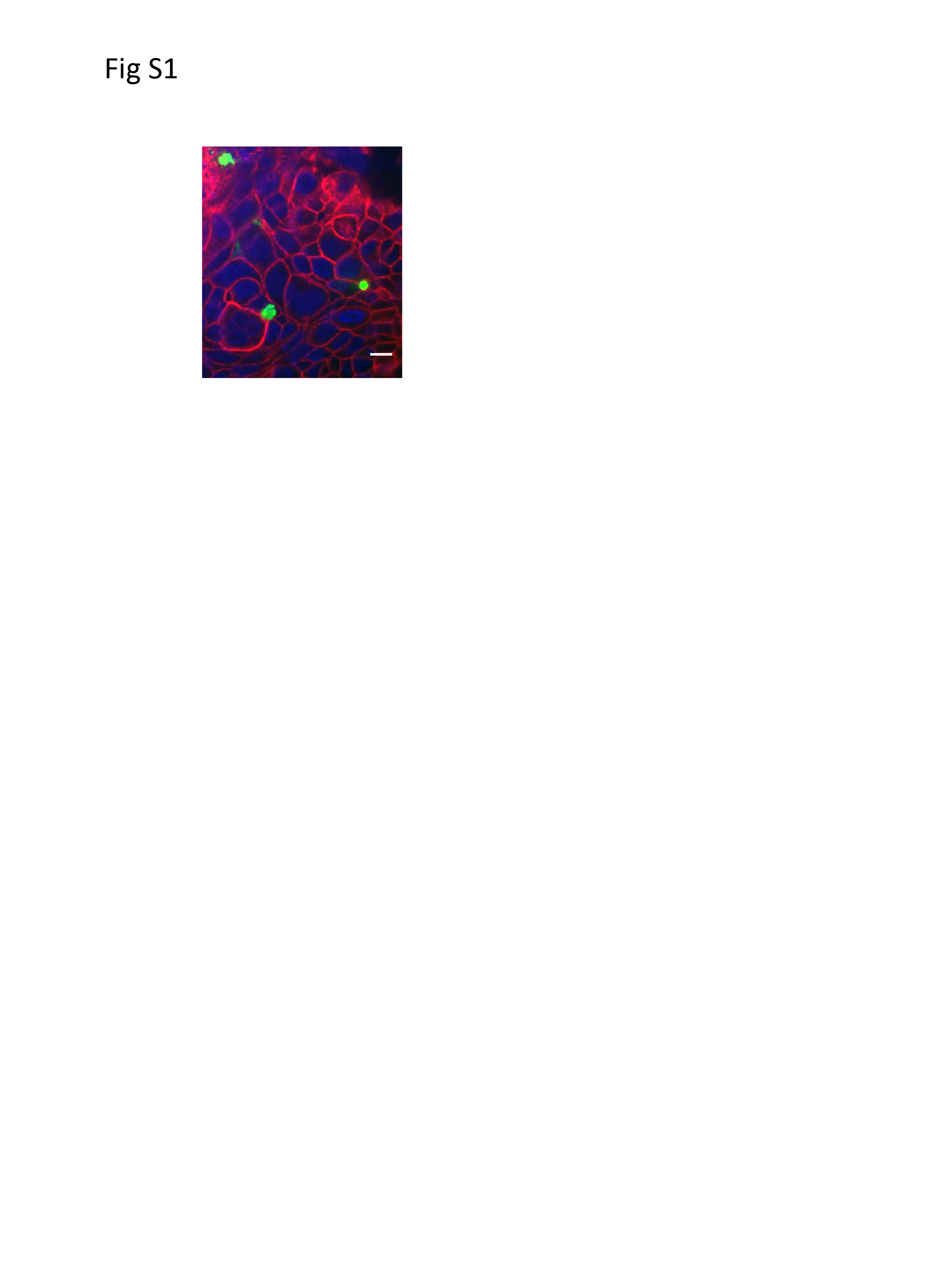

Supplement: FIGURE S1 — Characterization of mucus production in the Caco-2/HT29-MTX monolayer. Immunofluorescent microscopy images of Caco2/HT29-MTX monolayer (control, uninfected) stained with mucin 2 antibody showing mucus production (green) after 14 days of cell culture in the Snapwell insert. Actin is stained red and cell nuclei, blue. Scale bar = 10 μM. [file Image_1.TIF]

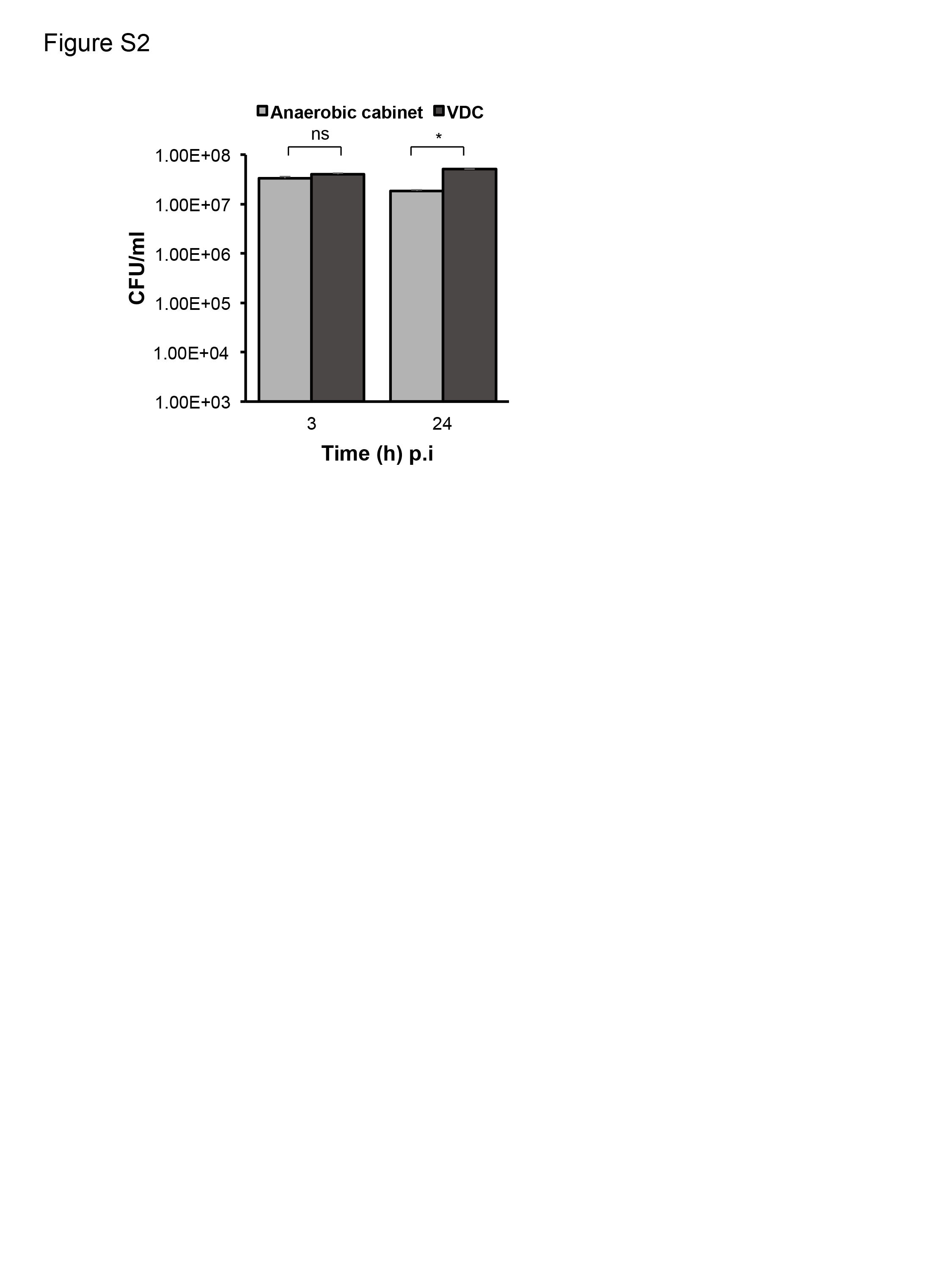

Supplement: FIGURE S2 — The vertical diffusion chamber maintains anaerobic conditions. Overnight cultures of C. difficile were centrifuged and equal numbers of bacteria (3.6 × 10^7^ CFU/ml) were resuspended in DMEM-10 and incubated in the VDC or anaerobic cabinet for 3 and 24 h. Serial dilutions of the apical supernatant were plated on BHI agar to enumerate colony counts. Data shown are the mean of three independent experiments and error bars indicate SD, *p < 0.05, ns, not significant by one-way ANOVA. [file Image_2.TIF]

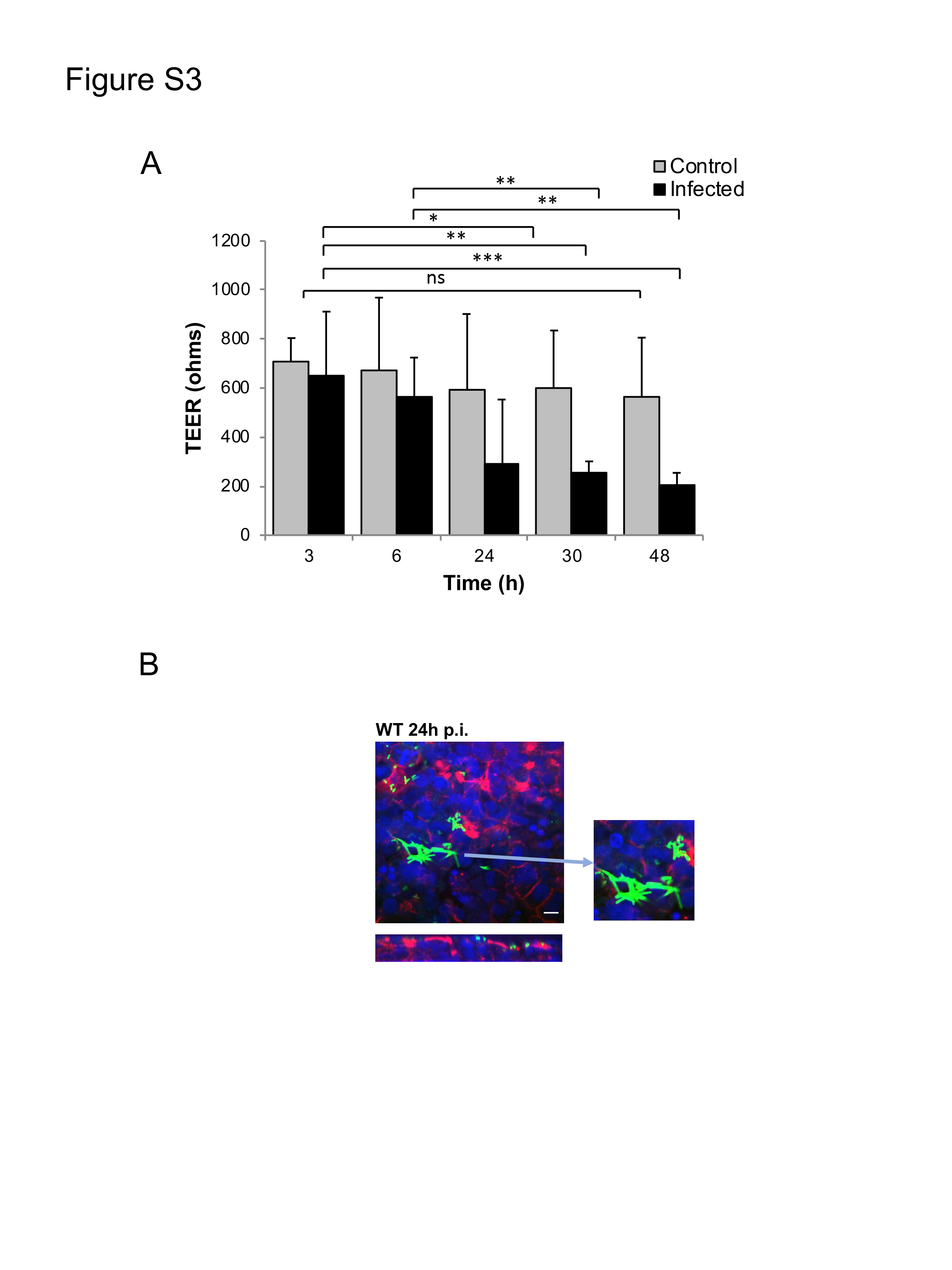

Supplement: FIGURE S3 — (A) Change in TEER during C. difficile infection. Reduction in TEER measurements at different times after infection. *p < 0.05, ∗∗p < 0.01, and ∗∗∗ p < 0.001 as determined by one-way ANOVA with Tukey’s test for multiple comparison. (B) Immunofluorescent microscopy image of C. difficile infected IECs at 24 h p.i. showing the bacteria stained with anti C. difficile antibodies (green) and actin stained with phalloidin (red). Cell nuclei are stained with DAPI (blue). Arrow indicates C. difficile present as micro-communities on the IECs. [file Image_3.TIF]

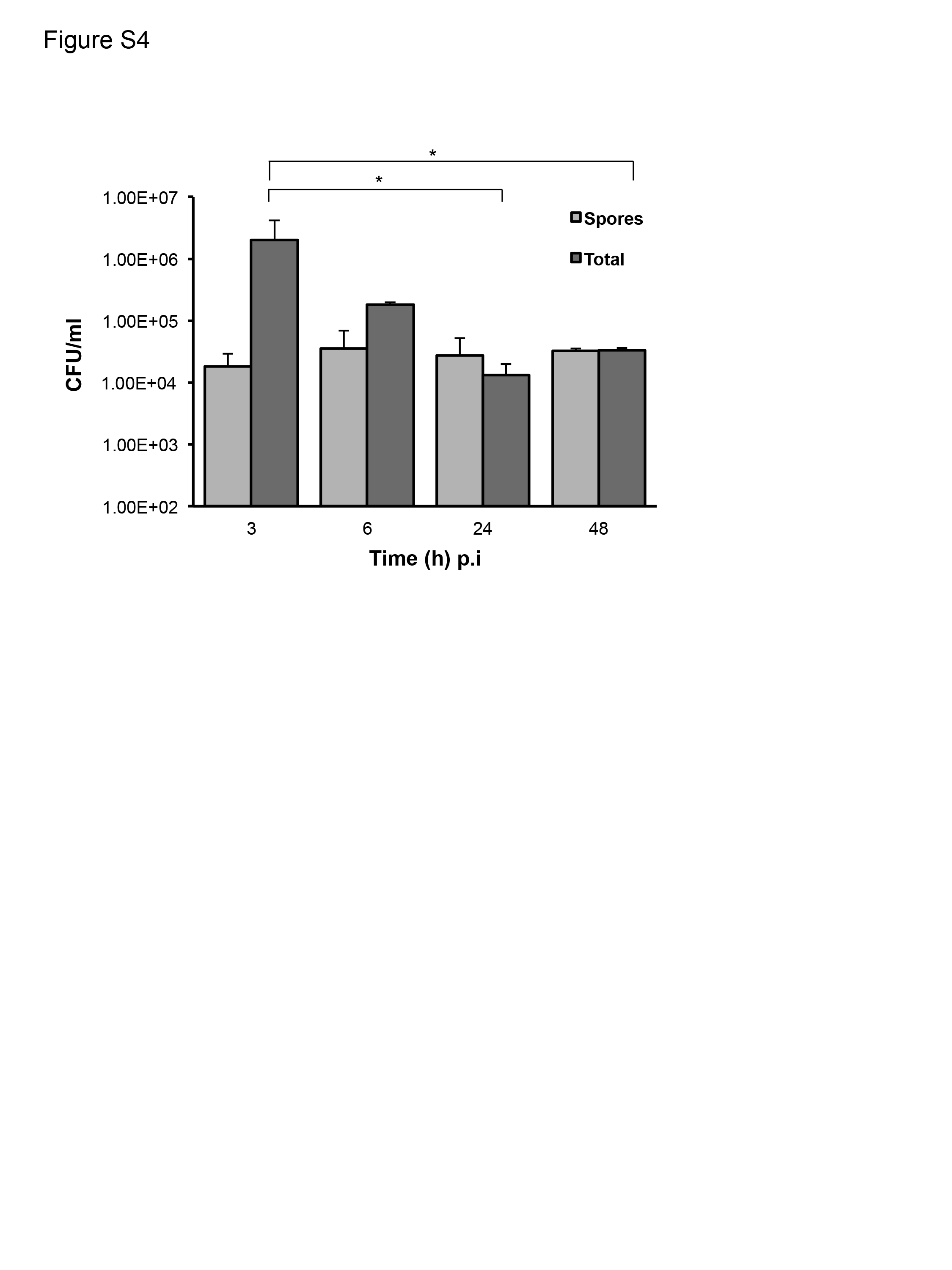

Supplement: FIGURE S4 — Spore counts from apical chamber culture supernatants from the E-VDC model. Colony counts of spores and total cells in the apical compartment supernatants. A significant decrease in total cell count was seen at 24 and 48 h p.i. but an increase in spores compared to the total bacterial numbers is observed. Data shown are the mean of three independent experiments and error bars indicate SD, *p < 0.05 as determined by two-way ANOVA. [file Image_4.TIF]

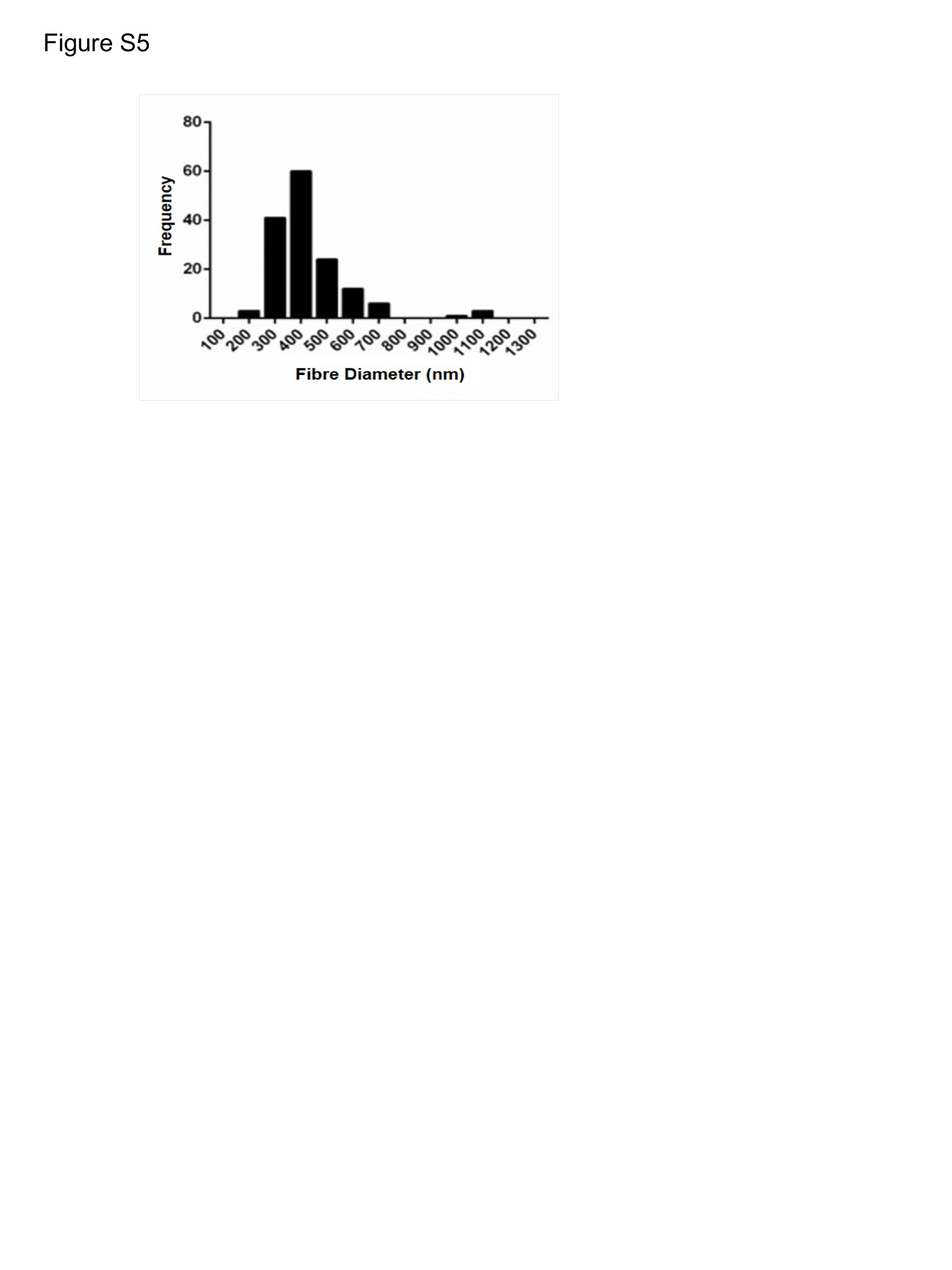

Supplement: FIGURE S5 — Fiber diameter of the electrospun nanofibrous matrix. Fiber diameter analysis revealed that the average fiber diameter was 457 ± 170 nm, with fibers in the range of 200–1100 nm (n = 150 measurements, 50 measurements per scaffold from three independently produced scaffolds). [file Image_5.TIF]

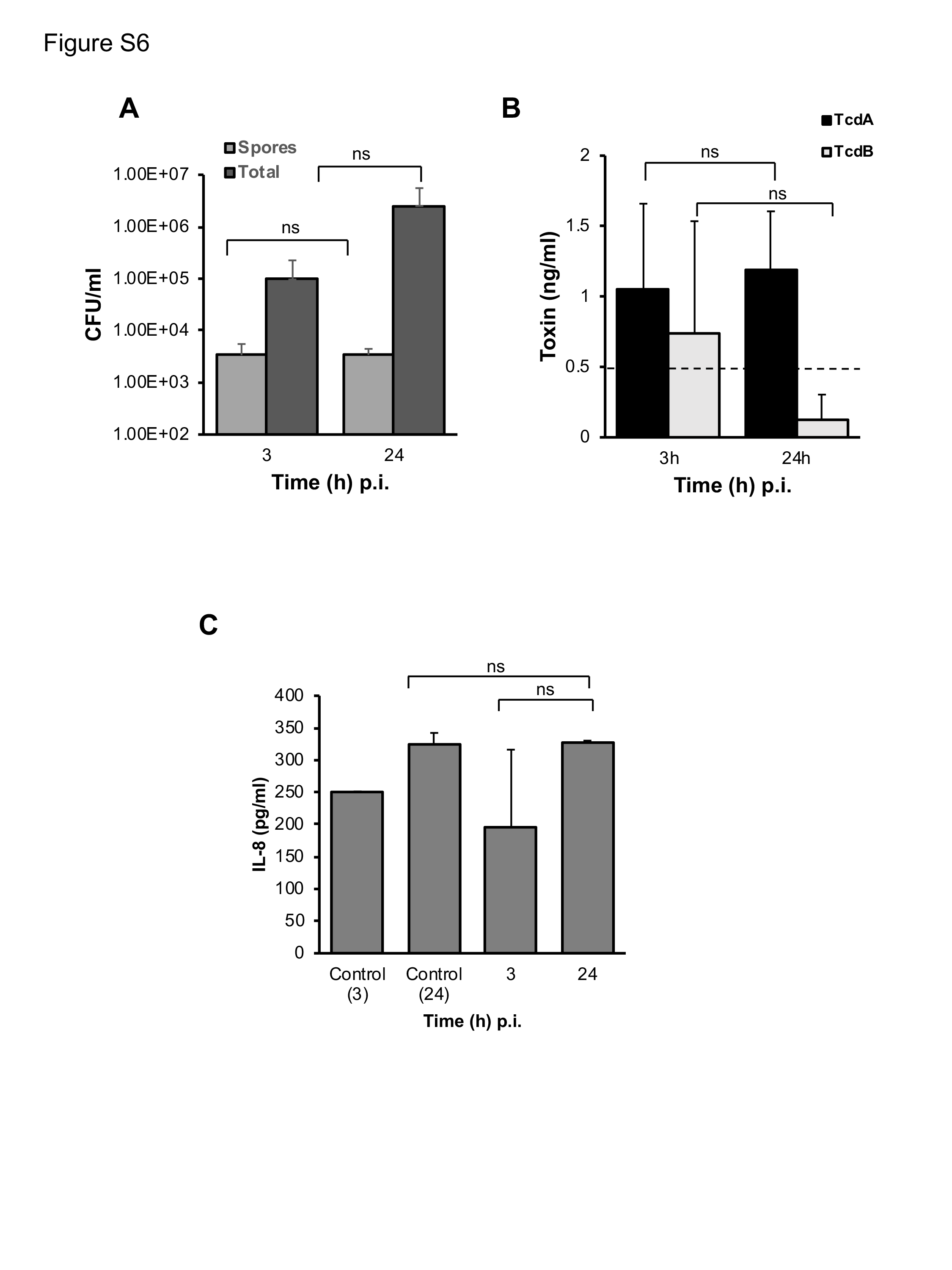

Supplement: FIGURE S6 — C. difficile spores and toxin production, and host response to infection in the M-VDC model. (A) Colony counts of spores and total cells in the host cell-associated C. difficile fraction of the M-VDC model. Data shown are the mean of three independent experiments and error bars indicate SD, ns, not significant as determined by two-way ANOVA. (B) Toxin A and B levels in the M-VDC model. Data shown are the mean of three independent experiments and error bars indicate SD, ns, not significant as determined by two-way ANOVA. Dashed line represents the sensitivity of the test at 0.5 ng/ml. (C) Human IL-8 levels in the M-VDC model. Data shown are the mean of three independent experiments and error bars indicate SD, ns, not significant as determined by the one-way ANOVA with Tukey’s test for multiple comparison. [file Image_6.TIF]

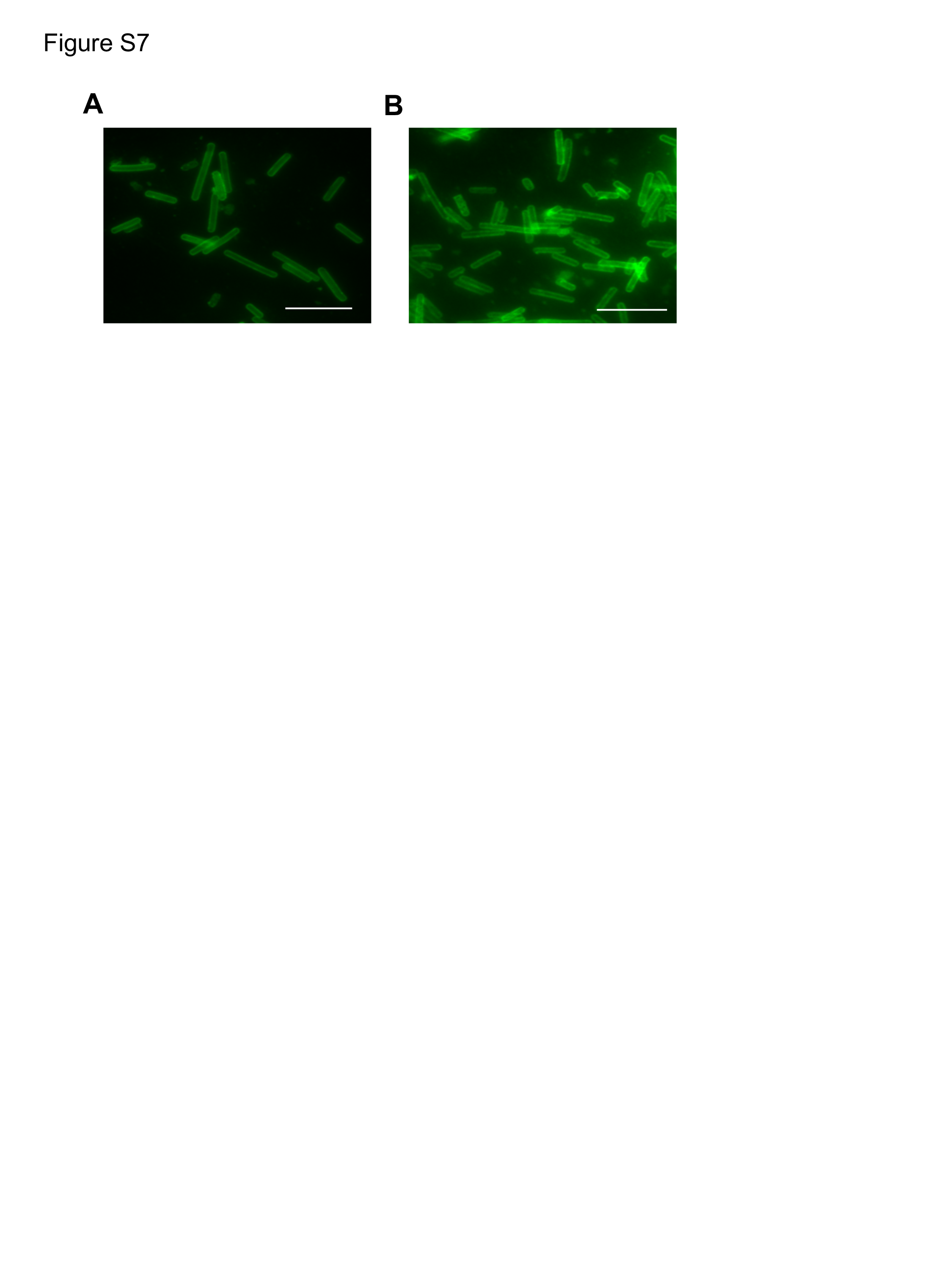

Supplement: FIGURE S7 — Filaments are not produced when C. difficile is grown for 48 h in culture in the absence of intestinal epithelial cells. (A) C. difficile was grown for 48 h using DMEM-10 and stained with anti-C. difficile for 1 h, followed by Alexa Fluor 488 goat anti-rabbit secondary antibody (green). (B) The basolateral supernatant (DMEM-10) from a 24 h infection in the VDC was used to grow C. difficile for 48 h to determine if cell released factors in the supernatant can induce filament formation. Staining with anti C. difficile for 1 h was performed, followed by Alexa Fluor 488 goat anti-rabbit secondary antibody (green). Images were taken with a Leica DMi microscope at 100x magnification. Scalebar = 10 μM. [file Image_7.TIF]
